# Cardiovascular Complications Secondary to Graves’ Disease: A Prospective Study from Ukraine

**DOI:** 10.1371/journal.pone.0122388

**Published:** 2015-03-24

**Authors:** Iryna Tsymbaliuk, Dmytro Unukovych, Nataliia Shvets, Andrii Dinets

**Affiliations:** 1 Department of Therapy, Shupyk National Medical Academy of Postgraduate Education, 04112 Kiev, Ukraine; 2 Department of Functional Diagnostic, Kyiv City Teaching Endocrinological Center, 01034 Kiev, Ukraine; 3 Department of Surgery #4, Bogomolets National Medical University, 01601 Kiev, Ukraine; 4 Department of Oncology-Pathology, Karolinska Institutet, 17176 Stockholm, Sweden; Uppsala University, SWEDEN

## Abstract

**Background:**

Graves’ disease (GD) is a common cause of hyperthyroidism resulting in development of thyrotoxic heart disease (THD).

**Objectives:**

to assess cardiovascular disorders and health related quality of life (HRQoL) in patients with THD secondary to GD.

**Patients and Methods:**

All patients diagnosed with THD secondary to GD between January 2011 and December 2013 were eligible for this study. Clinical assessment was performed at baseline and at the follow-up visit after the restoring of euthyroid state. HRQoL was studied with a questionnaire EQ-5D-5L.

**Results:**

Follow-up data were available for 61 patients, but only 30 patients with THD secondary to GD were consented to participate in investigation of their HRQoL. The frequency of cardiovascular complications was significantly reduced as compared before and after the antithyroid therapy as follows: resting heart rate (122 vs. 74 bpm), blood pressure: systolic (155 vs. 123 mm Hg), diastolic (83 vs. 66 mm Hg), supraventricular premature contractions (71% vs. 7%), atrial fibrillation (72% vs. 25%), congestive heart failure (69% vs. 20%), thyrotoxic cardiomyopathy (77% vs. 26%), all p<0.01. Anti-TSH receptor antibodies were determined as independent predictor of left ventricular geometry changes, (b-coefficient = 0.04, 95%CI 0.01–0.07, p = 0.02). HRQoL was improved in all domains and self-rated health increased from 43 to 75 units by visual analogue score (p<0.001).

**Conclusions:**

Restoring of euthyroid state in patients with GD is associated with significant elimination of cardiovascular disorders and improvement of HRQoL. To our knowledge this is the first study evaluating Ukrainian patients with THD secondary to GD with focus on HRQoL.

## Introduction

Graves’ disease (GD) is a common cause of hyperthyroidism constituting 1% of all endocrine disorders in Ukraine and 0.7–5% in other countries [1, 2]. GD is frequently associated with diffuse goiter, Graves’ ophthalmopathy, anti-TSH receptor antibodies (TRAb), antithyroid peroxidase antibodies (TPOAb) and high level of thyroid hormones thyroxin (T4) and *triiodothyronine* (T3) in serum [3]. An excess of thyroid hormones has a direct effect on the cardiac myocytes and peripheral vasculature, resulting in development of various cardiovascular complications classified as thyrotoxic heart disease (THD). THD could manifest as elevated resting heart rate, supraventricular premature contractions, atrial fibrillation, cardiac hypertrophy, arterial hypertension, thyrotoxic cardiomyopathy, congestive heart failure [4–7]. Moreover, thyroid hormones interact with neurotransmitters regulating mental activity, which can be deregulated due to hyperthyroidism resulting in development of psychiatric disorders [8, 9]. THD along with other complications of GD was shown to diminish health related quality of life (HRQoL) and associated with poor prognosis of the disease [10–12]. In general, patients with GD and hyperthyroidism demonstrate higher mortality mainly due to thromboembolism caused by atrial fibrillation [13].

The administration of antithyroid drugs and beta-blockers is initial treatment of GD resulting in elimination of hyperthyroidism and normalization of cardiovascular parameters as well as HRQoL [14–16]. It is worth to mention that HRQoL is well investigated in different patients with hyperthyroidism or cardiovascular disorders [10–13, 17, 18]. Available studies on HRQoL are mainly focused on the overall evaluation of GD, Graves’ ophthalmopathy, or report treatment modalities or certain cardiovascular complications. Thus, HRQoL in patients with THD secondary to GD remain controversial and little is known about the features of GD in Ukrainian population.

This prospective study aimed at evaluating HRQoL and cardiovascular changes in patients with THD secondary to GD.

## Patients and Methods

The study and consent procedure were approved by the ethical committee at Kyiv City Teaching Endocrinological Center (Kiev, Ukraine). All patients provided their written informed consent to participate in the study. All patients diagnosed with GD at the Kyiv City Teaching Endocrinological Center between January 2011 and December 2013 were eligible for the study. Among 2,221 individuals with hyperthyroidism, 1,194 patients were diagnosed with GD. Of these, 75 patients were presented with THD and invited to participate in the study. Clinical information and follow-up data were available for 61 patients, but only 30 patients with THD secondary to GD consented to participate in HRQoL part of the study.

The inclusion criteria were diagnosis of GD with overt hyperthyroidism and THD. We excluded patients diagnosed with cancer, infectious disease, lung, renal or liver failures, other types of secondary arterial hypertension, history of myocardial infarction or stroke, heart failures NYHA III and IV, congenital heart defects, valvular heart disease.

All patients underwent an antithyroid therapy with titration block-regimen by methimazole; metoprolol (beta-blocker), ramipril (angiotensin-converting enzyme (ACE) inhibitor) or telmisartan (angiotensin II receptor blocker) were administrated to treat cardiological complications (Table 1).

**Table 1:** Medications for treatment of cardiological complications before the antithyroid therapy and after the follow-up.

Drug	Type of action	Therapy at baseline			Therapy after follow-up	
		Daily dosage, mg, mean (range)	Nr of patients under therapy		Daily dosage, mg, mean (range)	Nr of patients under therapy
Metoprolol	Beta-1 adrenergic receptor blocker	70 (25–100)	61 (100%)		37 (12.5–50)	12 (20%)
Ramipril	Angiotensin-converting enzyme inhibitor	9 (5–40)	44 (72%)		4.4 (2.5–5)	12 (20%)
Telmisartan	Angiotensin II receptor blocker	40 (40)	2 (3%)		0	0

Patients were subjected to prospective evaluation: at baseline, *i*.*e*. at the time of GD diagnosis and at follow-up when the stable euthyroid state was established. Both assessments included HRQoL evaluation and clinical follow-up with relevant thyroid and cardiovascular examinations.

### HRQoL evaluation

HRQoL was evaluated using the Ukrainian printed version of the EQ-5D-5L questionnaire containing visual analogue score (VAS) system (www.euroqol.org EuroQol Group, Netherlands), previously validated in studies of cardiac disorders [19]. In a brief, patients reported their health status in five dimensions of the EQ-5D-5L (mobility, self-care, usual activities, pain/discomfort, anxiety/depression) each containing 5 levels of problems: no problem (level 1), slight (level 2), moderate (level 3), severe (level 4), or extreme problems (level 5). Then patients rated their overall health status in vertical VAS system with endpoints from 0 to 100 indicating worst and best imaginable health state, respectively. Obtained data were subsequently converted to index values as recommended elsewhere [20].

### The thyroid gland assessment

Diagnosis of GD was established for patients with biochemical evidence of overt hyperthyroidism, *i*.*e*. high levels of serum free T4 (normal ranges 11.96–22.01 pmol/l), free T3 (normal ranges 3.1–3.73 pmol/l), TSH < 0.1 mIU/l (normal ranges 0.27–4.2 mIU/l) accompanied by at least two of following parameters: diffuse goiter, Graves’ ophthalmopathy, increased level of TRAb (normal <1.58 IU/l), TPOAb (normal <34 kIU/l). The thyroid was evaluated by physical examination, blood testing (TSH, free T3, free T4, TRAb, TPOAb) by using Cobas E 411 analyzer (Roshe Diagnostics GmbH, Germany) and ultrasonography by using Sonoline Sienna equipped with 3.5–7.5 MHz transducers (Siemens, Germany).

### Cardiovascular evaluation

Resting heart rate was determined by manual evaluation of the heartbeats per minute (bpm) at the radial artery. Tachycardia was noted if resting heart rate was >100 bpm. THD was confirmed by an experienced cardiologist (IT) in patients with manifestations of supraventricular premature contractions, atrial fibrillation, arterial hypertension, thyrotoxic cardiomyopathy, or congestive heart failure according to available guidelines and recommendations [21–24]. Arterial hypertension was considered when systolic blood pressure ≥ 140 mm Hg or diastolic blood pressure ≥ 90 mm Hg. The office blood pressure (BP) level was measured three times on the right arm to determine the mean values by using manual BP monitor (Little Doctor, Singapore). Patients with a history of essential hypertension were regarded as having arterial hypertension secondary to GD in case of abnormal BP despite the anti-hypertensive therapy.

A 12-lead electrocardiography was performed by using electrocardiograph UCARD-200 (UTAC, Ukraine). Echocardiography was performed on the EnVisor C Ultrasound System (Philips, USA) with 2–4 MHz phase transducers using M-mode, two-dimensional imaging and Doppler technique to measure the following parameters of the heart: left ventricular (LV) mass and LV mass index, left atrium dimension, interventricular septum wall thickness, LV posterior wall thickness, LV relative wall thickness, LV end-systolic diameter, LV end-diastolic diameter, LV end-systolic volume and LV end-diastolic volume LV end-diastolic volume, LV end-systolic diameter, LV end-diastolic diameter, LV ejection fraction (LVEF); the pulmonary hypertension was diagnosed if BP in pulmonary artery was > 25 mm Hg as determined by Doppler-echocardiography; geometry of LV was determined by the comparison of LV relative wall thickness and LV mass index in male and female patients as recommended elsewhere [25]. Body mass index (BMI) was calculated by using formula: body weight in kg/(height in m^2^). Body surface area (BSA) was determined by Mosteller formula [26].

### Statistical analyses

The Statistica v12.0 (StatSoft Scandinavia AB, Sweden) software system was used for univariate statistical analyses. The Wilcoxon signed-rank test and Fisher’s (two-tailed) exact test were applied to evaluate the differences in the studied parameters. The level of statistical significance was set to p<0.01 due to multiplicity. A regression analysis was performed using STATA/SE v13 (StataCorp, TX, USA). In the linear regression, correlation of LV geometry changes was tested in the uni- and multivariate analyses with the following factors as age, BMI, systolic blood pressure, TSH, free T3, free T4, TRAb, TPOAb (all-continuous). Results with p<0.05 were considered as statistically significant.

## Results

### Clinical findings

Analyses of the clinical data of the cohort are summarized in Table 2. There were 46 (75%) women and 15 (25%) men with the mean age of 52 years (range 30–69). The mean follow-up time after the diagnosis was 6 months (range 4–14). The mean body weight at the time of diagnosis and after the antithyroid therapy was 74 kg and 76 kg, respectively, resulting in differences in BMI (25.3 vs. 26.6 kg/m^2^) and BSA (1.80 vs. 1.88 m^2^), p<0.001.

**Table 2:** Clinical characteristics of the patients before and after the antithyroid therapy.

Characteristics	Patients (n = 61)		p-value
	Before treatment	After treatment	
**Weight, kg**			
Mean, range	74 (50–115)	76 (52–117)	<0.001
Median	72	73	
**BMI, kg/m** ^**2**^			
Mean, range	25.3 (16.4–38.3)	26.6 (16.4–41.5)	<0.001
Median	24.2	25.5	
**BSA, m** ^**2**^			
Mean, range	1.8 (1.5–2.3)	1.88 (1.6–2.3)	<0.001
Median	1.85	1.87	
**TSH, mIU/l**			
Mean, range	0.023 (0.003–0.07)	2.8 (0.005–55.2)	<0.001
Median	0.005	2.2	
**fT3, pmol/l**			
Mean, range	27.4 (4.2–54)	5.2 (2–9.1)	<0.001
Median	24	5.4	
**fT4, pmol/l**			
Mean, range	81.3 (25.2–335)	17.4 (1.3–36)	<0.001
Median	78	16.2	
**TPOAb** *, **kIU/l**			
Mean, range	287 (5.4–6258)	117 (5–1018)	<0.001
Median	140	60	
**TRAb** **, **IU/l**			
Mean, range	104 (0.4–3457)	27 (0.4–305)	<0.001
Median	32	13	
**Volume of thyroid, cm** ^**3**^			
*Right lobe*			
Mean, range	49 (12–225)	67 (13–230)	<0.001
Median	31	52	
*Left lobe*			
Mean, range	45 (9–182)	62 (10–216)	<0.001
Median	32	45	
**Graves’ ophthalmopathy**			
Yes	42 (69%)	11 (18%)	<0.001
No	19 (31%)	50 (82%)	

* = missing in 6 cases

** = missing in 5 cases

### Elimination of hyperthyroidism

An ultrasonography at baseline detected the diffusely enlarged thyroid in 56 (92%) patients, measuring 49 cm^3^ (range 12–225 cm^3^) right lobe and 45 cm^3^ (range 9–182 cm^3^) left lobe. A significantly increased thyroid size was also detected after the treatment: 67 cm^3^ right lobe and 62 cm^3^ left lobe (p<0.001). At the baseline, Graves’ ophthalmopathy was present in 42 (69%) and in 11 (18%) patients after the antithyroid therapy (p<0.001). The hyperthyroidism was confirmed by low levels of TSH with mean concentration 0.023 mIU/l followed by its significant elevation to 3.6 μIU/ml after the antithyroid therapy (p<0.001). All patients exhibited overt hyperthyroidism followed by restoring of euthyroid state as judged from the thyroid hormones levels before and after the antithyroid therapy, respectively: free T3 (27.4 vs. 5.2 pmol/l), free T4 (81.3 vs. 17.4 pmol/l). These data indicate that decreasing of both free T3 and T4 in serum was supported by TSH elevation > 0.1 mIU/l in 48 (79%) cases after the treatment. Hence, a subclinical hyperthyroidism was diagnosed in remaining 13 (21%) patients without evidence of clinical manifestations.

Thyroid autoantibodies showed higher concentrations at the diagnosis followed by decreasing of their levels after the antithyroid therapy, respectively: TRAb (104 vs. 27 IU/l), TPOAb (87 vs. 117 kIU/l), p<0.001.

### Normalization of cardiovascular parameters

The evaluation of cardiac functions showed significant improvements of the hemodynamics, heart sizes and LV mass after the treatment (Table 3). The analyses of hemodynamics showed decreasing of resting heart rate from 122 to 74 bpm, systolic blood pressure from 155 to 123 mm Hg, diastolic blood pressure from 83 to 66 mm Hg in patients before and after the antithyroid therapy, respectively. The evaluation of cardiac chamber measurements demonstrated significant improvements of the studied parameters in all patients after the treatment, as follows: LV mass from 228 to 194 g, LV mass index from 129 to 107 g/m^2^, left atrium dimension from 4 to 3.7 cm, both interventricular and posterior septum wall thickness from 1.2 to 1.1 cm, LV relative wall thickness from 0.6 to 0.5 cm, LV end-systolic diameter from 3.6 to 3.2 cm, LV end-diastolic diameter from 5.2 to 4.7 cm, LV end-systolic volume from 51 to 42 ml, LV end-diastolic volume from 142 to 126 ml (p<0.001).

**Table 3:** Analyses of cardiovascular parameters before and after the antithyroid therapy in patients with Graves’ disease.

Parameters	Patients (n = 61)		p-value
	Before treatment	After treatment	
**Heart rate, bpm**			
Mean, range	122 (90–190)	74 (60–100)	< 0.001
Median	120	72	
**Systolic blood pressure, mm Hg**			
Mean, range	155 (130–190)	123 (70–155)	< 0.001
Median	155	120	
**Diastolic blood pressure, mm Hg**			
Mean, range	83 (60–120)	66 (50–80)	< 0.001
Median	80	65	
**Pulse blood pressure, mm Hg**			
Mean, range	72 (40–110)	58 (10–85)	< 0.001
Median	75	55	
**Left ventricular mass, g**			
Mean, range	228 (119–377)	194 (108–409)	< 0.001
Median	225	193	
**Left ventricular mass index, g/m** ^**2**^			
Mean, range	129 (65–229)	107 (59–229)	< 0.001
Median	122	101	
**Left atrium dimension, cm**			
Mean, range	4 (3.2–5.7)	3.7 (3.1–4.9)	< 0.001
Median	3.9	3.6	
**Interventricular septal wall thickness, cm**			
Mean, range	1.2 (0.9–1.4)	1.1 (0.9–1.9)	< 0.001
Median	1.1	0.6	
**Left ventricular posterior wall thickness, cm**			
Mean, range	1.2 (0.9–1.4)	1.1 (0.9–1.3)	< 0.001
Median	1.2	1.1	
**Left ventricular relative wall thickness, cm**			
Mean, range	0.6 (0.4–0.8)	0.5 (0.3–1.6)	< 0.001
Median	0.6	0.5	
**Left ventricular end-systolic diameter, cm**			
Mean, range	3.6 (2.7–4.8)	3.2 (2.6–5.1)	0.002
Median	3.6	3.2	
**Left ventricular end-diastolic diameter, cm**			
Mean, range	5.2 (4.2–6.2)	4.7 (3.8–5.8)	<0.001
Median	5.3	4.8	
**Left ventricular end-systolic volume, ml**			
Mean, range	51 (28–99)	42 (20–68)	<0.001
Median	52	40	
**Left ventricular end-diastolic volume, ml**			
Mean, range	142 (90–194)	126 (80–175)	<0.001
Median	147	126	
**Left ventricular ejection fraction, %**			
Mean, range	64 (46–79)	67 (56–81)	0.015
Median	64	66	
**Left ventricular geometry, n =**			
Normal geometry	1 (2%)	8 (13%)	0.032
Concentric remodeling	13 (21%)	20 (33%)	0.22
Eccentric hypertrophy	3 (5%)	9 (15%)	0.12
Concentric hypertrophy	44 (72%)	24 (39%)	<0.001

Further analyses of cardiovascular parameters showed higher frequency of cardiac rhythm disorders at the time of diagnosis than in euthyroid patients (Table 4), respectively: atrial fibrillation in 44 (72%) vs. 15 (25%) cases, supraventricular premature contractions in 43 (71%) vs. 4 (7%) cases (p<0.001). The thyrotoxic cardiomyopathy was diagnosed in 47 (77%) patients before and in 16 (26%) after the antithyroid therapy; congestive heart failure was diagnosed in 42 (69%) as compared to 12 (20%) patients (p<0.001).

**Table 4:** Evaluation of cardiovascular complications secondary to Graves’ disease before and after the antithyroid therapy.

Parameters	Patients (n = 61)		p-value
	Before treatment	After treatment	
**Arterial hypertension**			< 0.001
Yes	58 (95%)	1 (2%)	
No	3 (5%)	60 (98%)	
**Atrial fibrillation**			
Yes	44 (72%)	15 (25%)	<0.001
No	17 (28%)	46 (75%)	
**Supraventricular premature contractions**			
Yes	43 (71%)	4 (7%)	<0.001
No	8 (29%)	57 (93%)	
**Congestive heart failure**			
Yes	42 (69%)	12 (20%)	<0.001
No	19 (31%)	49 (80%)	
**Thyrotoxic cardiomyopathy**			
Yes	47 (77%)	16 (26%)	<0.001
No	14 (47%)	45 (74%)	
**Pulmonary hypertension**			
Yes	13 (21%)	3 (5%)	0.017
No	48 (79%)	58 (95%)	

### Improvement of LV geometry

All types of LV geometry were detected in study cohort (Table 3). A concentric hypertrophy was diagnosed in 44 (72%) of patients at baseline, while after the antithyroid therapy the frequency of this type of LV geometry was detected in 24 (39%) patients (p<0.001). Moreover, significant reversion of normal LV geometry was observed in 7 (11%) patients after the treatment (p = 0.032). These observations indicate the normalization of LV mass.

In the univariate analysis, TRAb was the only predictor of LV geometry changes, b-coefficient = 0.03 (95% CI 0.00–0.06), p = 0.032. The multivariate regression showed that association of TRAb and LV geometry changes remained statistically significant, b-coefficient = 0.04 (95% CI 0.01–0.07), p = 0.02. These data indicate that decreasing of TRAb levels is associated with LV geometry changes. The other factors did not show statistically significant association in the regression model (data not shown).

### Improvements of HRQoL after the antithyroid therapy

**Fig 1:**
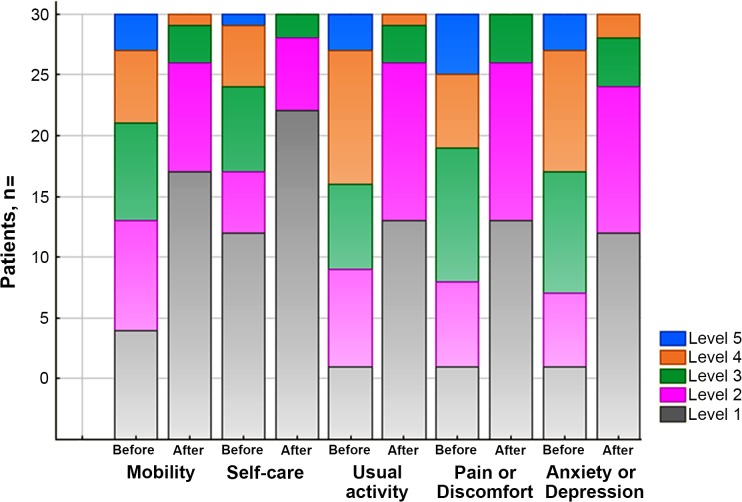
Illustration of HRQoL assessment by reported levels 1 to 5 for each dimension of the EQ-5D-5L instrument. Patients reported less problems after restoring of euthyroidism as compared to hyperthyroid state.

Analyses of the EQ-5D-5L data showed significant improvements of HRQoL in all domains (Table 5, Fig. 1). After the treatment, 13 (43%) patients reported elimination of problems in mobility dimension, which is 2 times less frequent as compared to 26 (86%) cases before the antithyroid therapy (p<0.001). We observed reducing of problems in self-care domain in 12 (77%) cases: 18 (60%) patients reported problems before as compared to 7 (23%) patients after the antithyroid therapy (p<0.001). Analyses of usual activity and pain/discomfort showed elimination of the problems in these two domains: 29 (97%) patients reported problems at baseline vs. 17 (57%) after the treatment (p<0.001). We observed a significant decrease in reported problems in anxiety/depression: 29 (97%) vs. 18 (60%) after the antithyroid therapy (p<0.01).

**Table 5:** Health related quality of life assessed by the EQ-5D-5L instrument.

	Patients (n = 30)		
Dimensions	Before treatment, n =	After treatment, n =	p-value
**Mobility**			
*Problems*	26 (87%)	13 (43%)	<0.001
*No problems*	4 (13%)	17 (57%)	
**Self-care**			
*Problems*	18 (60%)	7 (23%)	<0.001
*No problems*	12 (40%)	23 (77%)	
**Usual activity**			
*Problems*	29 (97%)	17 (57%)	0.001
*No problems*	1 (3%)	13 (43%)	
**Pain/Discomfort**			
*Problems*	29 (97%)	17 (57%)	<0.001
*No problems*	1 (3%)	13 (43%)	
**Anxiety/Depression**			
*Problems*	29 (97%)	18 (60%)	0.005
*No problems*	1 (3%)	12 (40%)	

**Fig 2:**
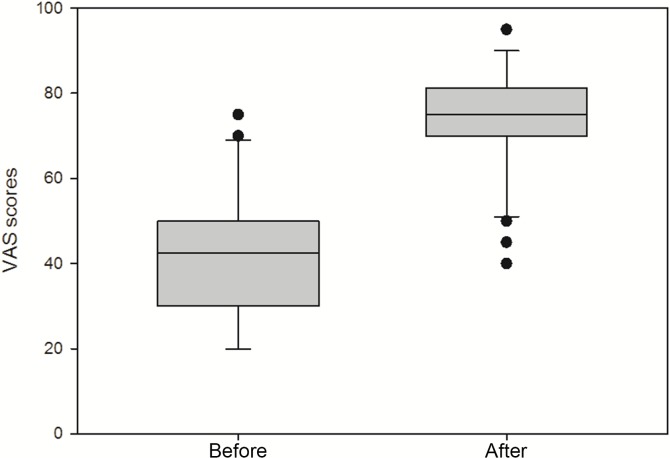
Graphical presentation of health status of patients by a Visual Analogue Scale (VAS). Box plots illustrating significantly lower self-rated health status of patients with hyperthyroidism (Before) as compared to euthyroid patients (After) by VAS scoring.

Self-rated health status is presented in Fig. 2. The VAS score has significantly improved after the therapy, 43 vs. 73 units (p<0.001), which is consistent with results obtained from the EQ-5D-5L instrument.

## Discussion

In this prospective study we investigated cardiovascular complications and HRQoL associated with hyperthyroidism in cohort of Ukrainian patients diagnosed with GD. Restoring of the euthyroid state was associated with elimination of cardiovascular disorders along with overall improvement of HRQoL.

We analyzed cardiovascular symptoms constituting THD secondary to GD in relation to HRQoL. In line with other researchers investigating relations between hyperthyroidism and cardiac disorders, we revealed a significant impact of GD on the development of cardiovascular complications followed by normalization of heart functions at the euthyroid state [1, 5, 9, 13, 27, 28]. Before the administration of antithyroid therapy, we detected elevated resting heart rate in all patients, which is commonly reported rhythm disorders in individuals with GD, possibly developing due to direct effect of thyroid hormones to the sinoatrial node [29]. Moreover, after euthyroidism restoration we observed reversion of supraventricular premature contractions and atrial fibrillation to sinus rhythm in majority of patients, which is in agreement with other reports [30–32]. In some studies, atrial fibrillation secondary to hyperthyroidism was reported as adverse factor associating with higher risk of CHF and thromboembolism [13, 27, 32]. Although we did not detect any embolic events, both atrial fibrillation and congestive heart failure were diagnosed in >50% of patients with hyperthyroidism along with impaired HRQoL, supporting the negative impact of these disorders on quality of life. Furthermore, before the antithyroid therapy we also diagnosed a thyrotoxic cardiomyopathy secondary to GD and remodeling of LV geometry, which is consistent with results of Oliveros-Ruiz et al [1]. We observed significant improvements of LV parameters, including reverting of concentric hypertrophy to other types of LV geometry. Moreover, our results are in agreement with studies of subclinical hyperthyroidism, suggesting the early detection and treatment of hyperthyroidism with antithyroid drugs is important factor for prevention and reversibility of thyrotoxic cardiomyopathy [1, 33].

We diagnosed arterial hypertension in 95% of patients, predominantly demonstrating high systolic blood pressure, as result of low vascular resistance, elevated resting heart rate and blood volume due to excess of thyroid hormones [29]. Moreover, we showed that arterial hypertension was developed secondary to GD and associated with diminished HRQoL. Restoring of euthyroid state resulted in elimination of arterial hypertension or stabilization of BP levels in patients with a history of arterial hypertension, which is consistent with other studies [34, 35].

The role of euthyroidism restoration is supported by findings from other studies showing the direct effect of hyperthyroid state on the cardiovascular system; in animal studies, the excess of thyroid hormones had a major impact on the cardiomyocytes, whereas beta-adrenergic or angiotensin receptor stimulations played a minor role [1, 29, 31, 36, 37]. Other studies demonstrated favorable effects of beta-blockers and ACE inhibitors on improvement of LV remodeling [38–40]. Hence, we hypothesize that improvement of cardiovascular parameters in relatively short follow up time was achieved after restoring of euthyroid state by antithyroid therapy accompanied by administration of beta-blockers and ACE inhibitors.

The thyroid autoantibodies TRAb and TPOAb were detected in majority of patients and TRAb was determined as the independent predictor of LV geometry changes. It is worth to mention that TRAb plays a role for prognosis of GD, in pathogenesis of Graves’ ophthalmopathy or development of pulmonary hypertension [41–43]. However, the impact of TRAb on the LV geometry is rather indirect, acting through the stimulation of thyroid hormones synthesis and their subsequent direct effect on the cardiomyocytes [5, 42].

Although the response rate for the EQ-5D-5L questionnaire was 49%, our results are consistent with other studies [2, 10–12, 29]. Analyses of physical dimensions of the EQ-5D-5L (“Mobility”, “Self-care” and “Usual activity”) showed decreasing of physical activity in hyperthyroid patients followed by significant improvement of these dimensions after treatment. The problems in physical dimensions are commonly reported in hyperthyroid individuals because of dramatic increase of cardiac work resulting in myocardial hypertrophy and low ability for adequate response to exercises [44, 45]. Furthermore, we showed high frequency of problems in pain/discomfort and anxiety/depression dimensions, which is also frequently demonstrated in patients with GD, other causes of hyperthyroidism and subclinical hyperthyroidism [8, 16, 46, 47]. The problems in pain/discomfort dimension can be developed due to diffuse goiter, Graves’ ophthalmopathy and cardiovascular complications [3, 4, 10, 31, 48]. Although the goiter size was increased after the antithyroid therapy, the normalization of thyroid hormones levels was associated with elimination of Graves’ ophthalmopathy in 30% of patients, stabilization of hemodynamic parameters and subsequent decreasing of the problems in majority of patients, which is in agreement with other reports [8, 16, 29].

Similarly to other studies, we showed that elimination of hyperthyroidism was associated with significant reduction of anxiety and depression [25, 29, 48, 49]. It is worth to mention that these two mood disorders are commonly reported in hyperthyroid patients due to pathophysiological interplay between the excess of thyroid hormones and neurotransmitters regulating mental health *conditions* [8, 9]. Moreover, anxiety and depression symptoms are commonly overlapped and linked to development of adverse cardiovascular events as showed in current study and supported by results of other investigations [14–16]. The anxiety and depression demonstrate a negative impact on HRQoL in patients with THD, but restoring of euthyroid status eliminates these mood disorders. Further analyses of HRQoL by using VAS technique revealed significant improvement of overall self-rated health status in all patients after the antithyroid therapy, which is consistent with our findings from the EQ-5D-5L.

The limitation of the study is a relatively small sample size, which could have a potential impact on the external validity. However, it is important to note that our results were in agreement with findings from the studies evaluating larger cohorts of patients [1, 13, 31, 50]. Moreover, the similar limitations are also seen in other studies, indicating the overall problem with the sample collection [27, 28, 30, 34, 41, 51].

To summarize, we reported the results of prospective study investigating THD secondary and HRQoL in patients with GD. The data analyses revealed the impact of hyperthyroidism to patients’ health status followed by significant improvement of HRQoL and elimination of cardiovascular complications constituting THD after the normalization of thyroid hormones levels in majority of patients, which are partly overlapping and partly distinguishing from other reported cohorts. To our best knowledge this is the first study from Ukraine evaluating HRQoL by the EQ-5D-5L instrument in patients with THD secondary to GD.
